# Cod-Liver Oil

**Published:** 1849-04

**Authors:** 


*Cod Liver Oil.*—H. B. Skinner, M. D., 60½ Cornhill, Boston, is a manufacturer of this now much-admired remedy. He is successful in overcoming the offensive fishy odor, which is one of the formidable obstacles with some, to its internal administration. In transparency and freedom from impurities, specimens from this establishment are quite unobjectionable, and the demand for a pure article is constantly increasing. Those embarked in the enterprise of furnishing the medical market must reap a rich harvest.

Mr. Souther, in Leverett street, has also acquired reputation for the elegant manner of putting up the oil of his make. Till he gave us some statistics in regard to the extent of this comparatively new business in Boston, we had no conception of the active demand for cod liver oil. Mr. Souther, in the first place, has quite a squadron of fishermen under his patronage, who are deriving an unexpected revenue from a source never contemplated by them till this new enterprise was started. Mr. Souther manufactures hundreds upon hundreds of gallons, in a short space of time—and to keep his presses working, he is receiving daily the raw material, sometimes as many as ten barrels a day, of the livers of cod-fish, from which alone the true medicinal cod-liver oil is procured. Boxes of beautifully labelled bottles are sent off in all directions; and, although considerable competition already begins to be exhibited, the supply from all reliable sources is represented to be inadequate to meet the influx of orders from abroad.

An oil is also obtained from the skate, ling, cusk, and some other fishes on this coast; but less confidence is reposed in either of these oils than in that from the cod. This oil consists chiefly of oleic and margaric acids, in union with glycerin, combined with a small proportion of resinous matter, phosphoric acid, iodine, and bromine. Its medical properties are ascribed to the iodine, small as the quantity is in any given quantity.

In strumous diseases, much reliance is placed on its action. Its flattering influences are fully admitted by taking a few ounces habitually; and the complexion, too, is represented to become clear and much improved by persisting in its use. This fact may ultimately lead to its patronage among ladies, as a cosmetic.—*Bost. Med. and Surg. Jour.*

